# Genome-wide identification and expression analysis of xyloglucan endotransglucosylase/hydrolase genes family in Salicaceae during grafting

**DOI:** 10.1186/s12864-023-09762-y

**Published:** 2023-11-09

**Authors:** Le Yang, Yao Chen, Xuejiao Liu, Sheng Zhang, Qingquan Han

**Affiliations:** 1 Key Laboratory of Bio-Resource and Eco-Environment of Ministry of Education, College of Life Sciences, Sichuan University, Chengdu, 610065 China; 2 The Engineering Research Institute of Agriculture and Forestry, Ludong University, 186 Hongqizhong Road, Yantai, 264025 Shandong Province China

**Keywords:** Poplar, Willow, *XTH* family, Graft, Genome identification

## Abstract

**Background:**

Poplar (*Populus cathayana*)and willow (*Salix rehderiana*) are important fast-growing trees in China. Grafting plays an important role in improving plant stress resistance and construction of ornamental plants. It is found that willow scions grafted onto poplar rootstocks can form ornamental plants. However, this grafted combination has a low survival rate. Many studies have reported that the xyloglucan endotransglucosylase/hydrolase (*XTH*) family plays an important role in the healing process of grafts.

**Results:**

A total of 38 *PtrXTHs* and 32 *SpuXTHs* were identified in poplar and willow respectively, and were classified into three subfamilies. Tandem duplication was the main reason for the expansion of the *PtrXTHs*. Grafting treatment and Quantitative real time PCR (RT-qPCR) analysis revealed that five *XTH* genes differentially expressed between self-grafted and reciprocal grafted combinations. Specifically, the high expression levels of *SrXTH16*, *SrXTH17*, *SrXTH25*, *PcXTH22* and *PcXTH17* may contribute to the high survival rate of the grafted combination with willow scion and poplar rootstock. Subcellular localization identified that the SrXTH16, SrXTH17, SrXTH25, PcXTH17 and PcXTH22 proteins were located on the cell walls. Transcription factors (NAC, MYB and DOF) may regulate the five *XTH* genes.

**Conclusions:**

This study provides a new understanding of the roles of *PcXTH* and *SrXTH* genes and their roles in grafting. Our results will give some hints to explore the molecular mechanisms of *PcXTH* and *SrXTH* genes involved in grafting in the future.

**Supplementary Information:**

The online version contains supplementary material available at 10.1186/s12864-023-09762-y.

## Background

Salicaceae plants have about 50 genera, *e.g*., *Populus*, *Chosenia* and *Salix*, with ~ 1000 species all over the world [1]. *P. cathayana* and *S. rehderiana* are fast-growing native species and have important ecological value in China [2–4]. As for their strong adaptability and cold resistance, the two species have been the most intensive plantation species, and widely distribute in the eastern Tibetan Plateau [5, 6]. Grafting can produce plants with good characteristics, and is widely used in horticulture, agriculture and forestry. Compared to intra-specific grafted plants, inter-specific grafted plants are usually more difficult to obtain and show greater heterosis [7]. Recently, we have successfully performed the inter-generic grafting between *Populus* and *Salix*, in which the combination of willow as scion and poplar as rootstock is the highest survival rate [8]. However, it is still unknown what the molecular mechanisms of the different graft survival rates.

In recent years, research on the grafted healing site has become a research hotspot. The graft healing process has been identified as five stages, including cambial tissue arrangement, isolation layer appearance, callus formation, cambium restoration, and connective tissue connection [9]. New vascular formation is the key point for successful grafting [9, 10]. The WUSCHEL-related homeobox 4 (WOX4), wound induced dedifferentiation 1/2/3 (WIND1/2/3), and WOX13 have been shown to play important roles during vascular formation process in the grafted plants [11]. RNA-seq analyses also indicated that cell wall-related genes played significant roles in promoting plant grafting healing [12, 13]. It is believed that cell wall modification/reconstruction enzymes positively participate in the entire grafting process to regulate new cell wall establishment and expansion [14, 15].

Cell wall can protect cell from changing internal environment and it determines the cellular shape and size of cells during plant development [16, 17]. The cellulose, hemicellulose, pectin, and glycoproteins are the main components of the primary cell wall [16, 18]. Xyloglucan is an abundant hemicellulose in the primary cell wall that not only associates with cellulose formation, but also promoting the linkage of the main load-bearing network with pectic network in the primary cell wall [16, 18, 19]. Many studies have indicated that the xyloglucan endotransglucosylase/hydrolase (XTH) family, which cleaves and reconnects xyloglucan molecules, is responsible for cell wall synthesis and reconstruction [16]. Cell wall reconstruction is an important basis for vascular differentiation in dicotyledonous plants [19]. Therefore, *XTH* genes may play key roles in the healing process of grafts [16, 18].

The *XTH* family is the subfamily of glycoside hydrolase family (GH16) [17]. It has been identified through genome sequencing in multiple plants, *e.g*., maize (*Zea mays*), napa cabbage (*Brassica rapa*), tobacco (*Nicotiana tabacum*), rice (*Oryza sativa*) and apple (*Malus pumila*) [20–24]. In *Arabidopsis*, 33 *XTH* genes have been identified and classified into three groups (I/II, III, and ancestral group) [16]. According to the different functions of the encoded proteins, the group III was subdivided into III-A and III-B groups [16]. Due to the two effects on xyloglucan chains, *XTH* encoded enzymes can be divided into two types [17]. Genes involved in the I/II and III-B groups encode xyloglucan endohydrolase (XET) enzyme, which cleave old chains and reconstruct new ones [25]. Genes in the III-A group encode xyloglucan endoglucosidase (XEH) enzyme, which hydrolyses xyloglucan chains, resulting in cell wall expansion and degradation [16, 17, 26].

*XTH* plays a crucial role in plant wound repair and grafting. A study has found that NAC domain containing protein (NAC071) can promote the expression of *XTH19* and *XTH20* at the wound of inflorescence stem [27]. The two genes have functional redundancy, both can promote wound healing by promoting the proliferation of marrow cells [27]. In some grafting experiments, it was found that the up-regulated expression of *XTH* gene was related to promoting successful grafting [11, 28]. In this study, we identified the whole genome of the *XTH* gene family and summarized their physical and chemical properties in poplar and willow. The evolutionary relationship of *XTH* family was analyzed in poplar and willow. Meanwhile, the gene expression pattern of *XTH* family genes during graft healing process was performed. Additionally, transcription factors that might regulate *XTH* genes were predicted.

## Results

### Identification and characteristics of PtrXTH and SpuXTH

There are 32 SpuXTHs and 38 PtrXTHs obtained in willow and poplar, respectively (Table S1). They are named SpuXTH1-SpuXTH32 in willow and PtrXTH1-PtrXTH38 in poplar. We also predicted the characteristics of SpuXTH and PtrXTH proteins (Table S1). The length of XTH proteins was about 241-374 amino acids in willow and 285-351 amino acid in poplar. The lowest number of amino acids in willow XTH protein is lower than that in poplar XTH protein, and the largest number is higher than that in poplar. The molecular weight (MW) was about 27,521-42,804 KDa in willow and 25776-40771 KDa in poplar. The minimum and maximum MWs of XTH protein in willow are higher than those in poplar. The isoelectric point (pI) was about 4.90-9.51 in willow and 4.61-9.47 in poplar. Understanding the pI of protein is of great significance for precipitation, extraction and purification of plant proteins and further structural and functional research. The instability index ranged from 27.90 to 56.21in willow and from 27.44 to 55.69 in poplar. This provides a reference for the application of XTH protein in other fields. Finally, the aliphatic index was 57.95-75.42 in willow and 56.28~76.61 in polar, which indicated that they were all hydrophilic proteins and soluble in water. 

### Chromosome mapping and collinearity analysis of *XTHs*

**Fig. 1:**
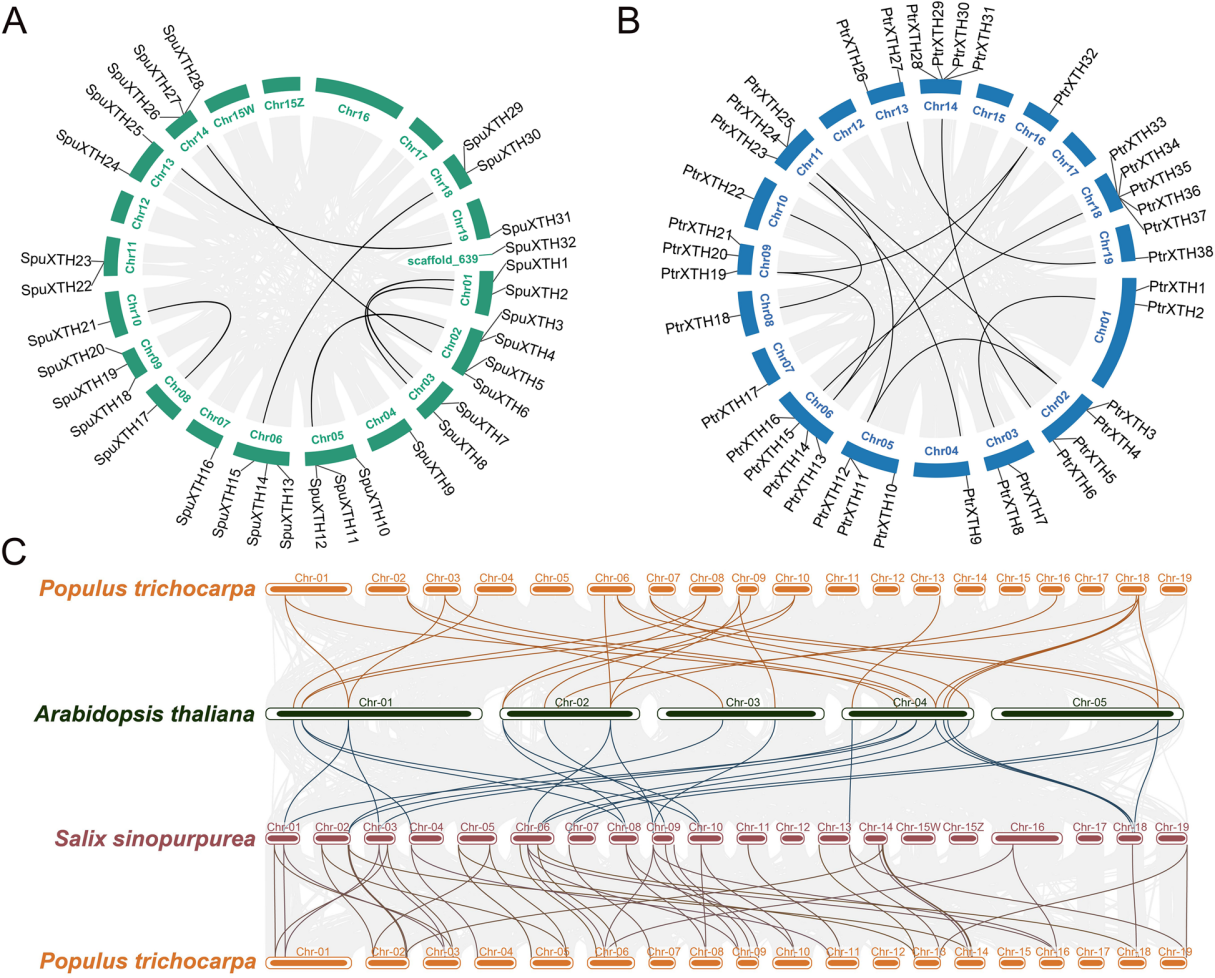
The collinearity analysis of *Populus*, *Salix* and *Arabidopsis*. Firstly, the collinearity analysis within species were conducted, including *Salix* (**A**) and *Populus* (**B**). Then the collinearity analysis among species were conducted, including *Populus* and *Arabidopsis*, *Salix* and *Arabidopsis*, and *Populus* and *Salix* (**C**). The gray lines in the background represent collinear blocks between genomes of species, while the black, orange, blue and red lines highlight the syntenic *XTH* gene pairs

Intraspecies collinearity analysis showed the chromosomal distribution of the *SpuXTH*s and *PtrXTH*s, which was determined by previous evolutionary events [29] (Fig. 1A, B). The *SpuXTH32* was distinctively distributed on one chromosome segment due to the incomplete genome assembly. The rest of the *SpuXTHs* were irregularly distributed on the chromosomes except Chr12, Chr15W, Chr15Z, Chr16 and Chr17. *PtrXTHs* were sporadically located on the chromosomes except Chr12, Chr15 and Chr17. The maximum number of *XTH* genes on one chromosome was higher in poplar than in willow, and the minimum number was equal.

The gene duplication, a force of gene expansion and evolution, is usually be divided into two situations, the tandem duplication and the segmental duplication. Tandem duplication is usually found in gene clusters to uncover the expansion of gene families [30]. We found three gene clusters in *Salix*, located on chromosomes 02, 05, and 11, respectively, including six *XTH* genes (*SpuXTH13*-*14*, *SpuXTH11*-*12*, *SpuXTH22*-*23*) (Fig. 1A). Four gene clusters, *PtrXTH3*-*4*, *PtrXTH11*-*12*, *PtrXTH24*-*25* and *PtrXTH34*-*37*, were respectively located on Chr02, Chr05, Chr11 and Chr18 in *Populus* (Fig. 1B). Inter-species collinearity analysis revealed that there were 12 segmental duplication events which involved 24 genes in poplar and 7 which contained 14 genes in willow. These gene pairs were used to calculate Ka (synonymous substitution)/Ks (non-synonymous substitution) values to assess the evolutionary selective pressure on *XTH* genes (Table S2). Results showed that 18 *XTH* gene pairs were detected with the Ka/Ks < 1, which suggested that these genes have suffered strong purifying selection. The sequences of *PtrXTH12* and *PtrXTH24* are highly diverge and have a long evolutionary distance, thus the Ka/Ks value is null.

To further analyze the evolution of *XTH* genes in plants, we conducted collinearity analysis in *Salix*, *Populus* and *Arabidopsis* (Fig. 1C). Syntenic maps revealed that there were 50 pairs of homologous genes between *Salix* and *Populus*, 24 pairs of homologous genes between *Salix* and *Arabidopsis*, and 26 pairs of homologous genes between *Populus* and *Arabidopsis*. Thus, there is a closer homology of *XTH* genes in *Salix* and *Populus* than those in *Arabidopsis* with *Salix* or *Populus*.

### Phylogenetic analysis and classification of XTH proteins

**Fig. 2:**
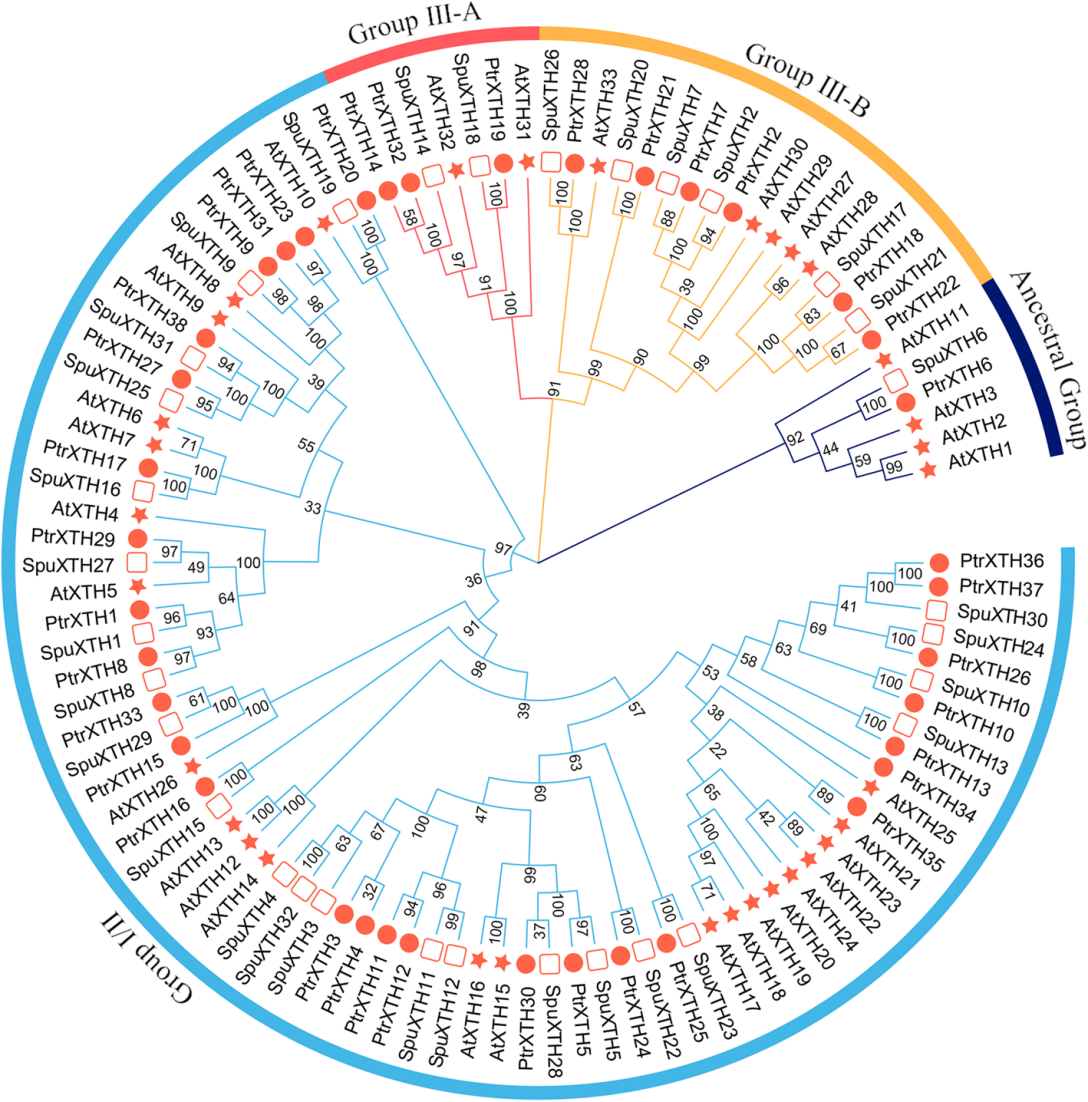
Evolutionary and phylogenetic analysis of the *XTH* genes family in *Populus*, *Salix* and *Arabidopsis*. The bootstrap values were listed in the evolutionary tree. Different shapes represented different species. The red circle represented *Populus*. The red box represented *Salix*. The red five-pointed star represented *Arabidopsis*. The colors of the outer ring and branches indicated different group

To elucidate the evolutionary relationship, we used multiple sequence alignment to analyze the 103 full-length XTH protein sequences from *Salix*, *Populus* and *Arabidopsis*. The neighbor-joining method was used to construct the phylogenetic tree (Fig. 2). The phylogenetic tree consists of three predominant branches. 73 proteins are classified into the largest I/II group. Within this group, 23 XTH proteins belonged to willow and 28 belonged to poplar. The III group can be subdivided into III-A and III-B groups. The phylogenetic analysis indicated that SpuXTH14, SpuXTH18, PtrXTH14, PtrXTH19 and PtrXTH32 belonged to the III-A group, in which these genes had XEH activity. SpuXTH2, SpuXTH7, SpuXTH17, SpuXTH20, SpuXTH21, SpuXTH26, PtrXTH2, PtrXTH7, PtrXTH18, PtrXTH21, PtrXTH22 and PtrXTH28 were grouped into III-B group, which mainly exhibited XET activity. The ancestral group had the fewest XTH proteins, including AtXTH1, AtXTH2, AtXTH3, AtXTH11, SpuXTH6 and PtrXTH6.

### Gene structure and conserved motifs of XTH

**Fig. 3:**
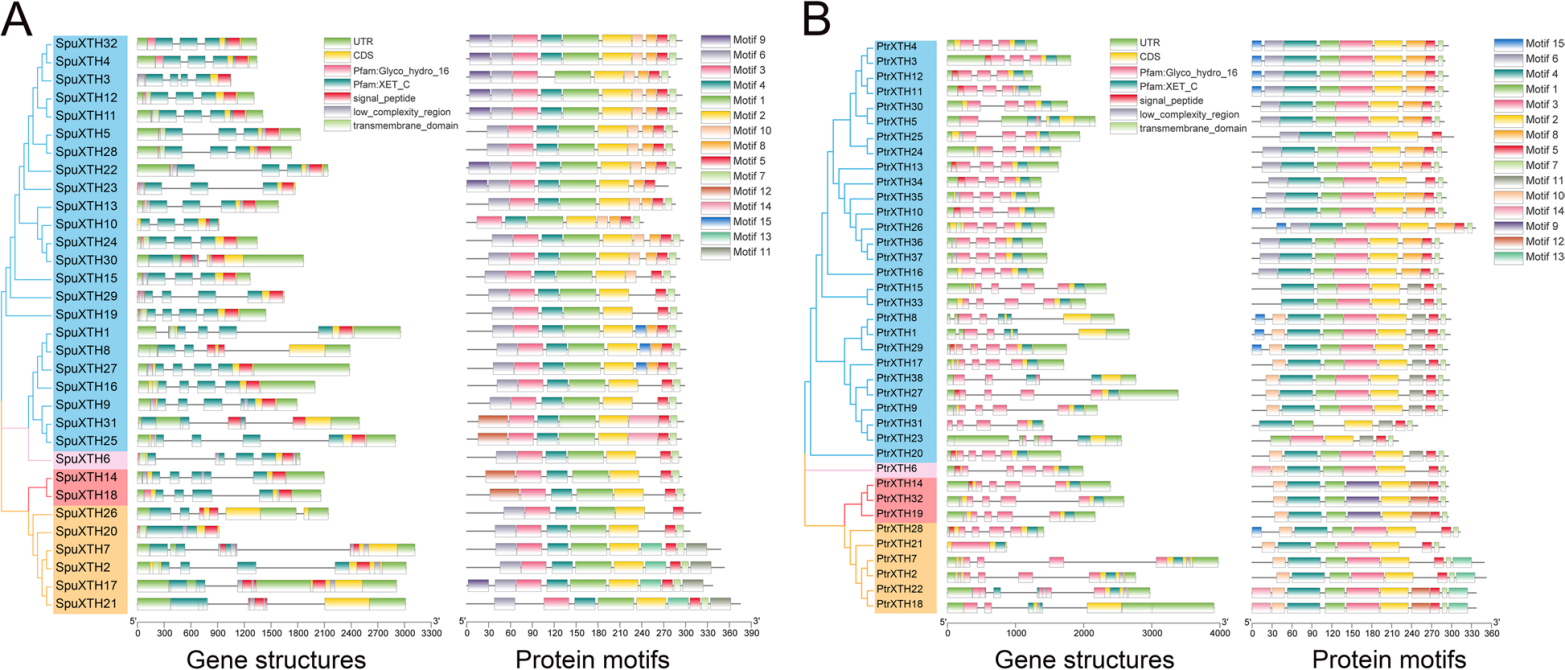
Phylogenetic and motifs analysis of PtrXTH and SpuXTH proteins, and gene structure and conserved domain structure analysis of *PtrXTH* and *SpuXTH* genes. **A** Phylogenetic tree and protein motifs were showed using full-length amino acid sequences from SpuXTHs. Blue represents I/II group, pink represents ancestral group, red represents III-A group and orange represents III-B group. Exon–intron structures and conserved domains were analyzed using gene sequences of *SpuXTHs*. **B** Phylogenetic tree and motifs were showed using full-length amino acid sequences from PtrXTHs. Blue represents I/II group, pink represents ancestral group, red represents III-A group and orange represents III-B group. Exon–intron structures and conserved domains were analyzed using gene sequences of *PtrXTH* genes

To gain further insight into the structural diversity of the *XTH* genes in poplar and willow, the structure of the 32 *SpuXTHs* and *38 PtrXTHs* was identified using TBtools software based on genomic sequences (Fig. 3). The number of exons varied from one to five in willow and from one to four in poplar (Fig. 3). *SpuXTH20* and *PtrXTH21* have no intron and untranslated region (UTR). *SpuXTH3*, *SpuXTH10*, *SpuXTH23*, *SpuXTH29* and *PtrXTH31* have no UTR either.

Glyco_hydro_16 and XET_C are conserved domains located on the *XTH* (Fig. 3). Most of the *SpuXTH* and *PtrXTH* proteins contained a putative signal peptide, which was a short peptide that is the key for translocation to the cellular membrane or extracellular space [31]. Additionally, the low_complexity_region, which could expand protein sequence space was identified in 24 *XTH* genes in willow and 15 *XTH* genes in poplar [32]. The transmembrane_domain, comprising a stretch of 17-25 hydrophobic amino acid residues that are structured as an α-helix, was found in 18 *SpuXTH* genes and 2 *PtrXTH* genes [33].

The conserved motifs were predicted as well (Fig. 3). Almost all XTHs in the same group shared common motifs. Motifs 1-3 and motif 5 were highly conserved in all SpuXTHs, while motifs 1-2, and motif 5 were highly conserved in all PtrXTHs. They were possibly composed of specific conserved domains Glyco_hydro_16 and XET_C. The proteins had six to ten motifs in willow and poplar. Some I/II group proteins in willow had motif 6 and motif 9, while other proteins in I/II group did not have. The III-B group proteins had the same motifs, except for SpuXTH17. The III-A group was consisted of SpuXTH14 and SpuXTH18, which had XEH activity, probably because they lacked motif 6 and motif 14. The motifs of PtrXTHs in the I/II group were multiple, indicating that these proteins have different functions. Except for PtrXTH23 and PtrXTH31, the rest of the genes in I/II group had motifs 1-5. Additionally, proteins in I/II group either had motif 6 or motif 11. The proteins of poplar in III-B group shared the same motifs, which indicated that the encoded proteins had similar structure and function. The PtrXTH proteins in the III-A group had a special motif 9 that wasn’t identified in other groups, which might be the reason why they had XEH activity.

### Structure-based sequence alignment and prediction of protein tertiary structure

**Fig. 4:**
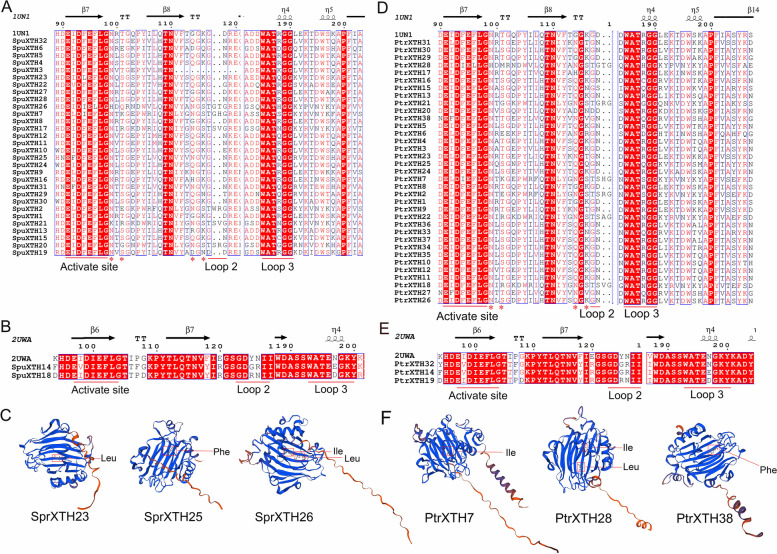
Structure-based sequence alignment of SpuXTH and PtrXTH proteins. **A** The SpuXTH proteins of Group I/II, III-B and ancestral group. **B** The SpuXTH proteins of III-A group. **C** The tertiary structure of SpuXTH6, SpuXTH14 and SpuXTH25. **D** The PtrXTH proteins of Group I/II, III-B and ancestral group. **E** The PtrXTH proteins of III-A group. **F** The tertiary structure of PtrXTH6, PtrXTH19 and PtrXTH26. The secondary structures of α-helices (spiral), β-sheets (arrows) and N-glycosylation site (*) are indicated. Leu is short for leucine. Phe is short for phenylalanine. Ile is short for isoleucine

The proportion of XTHs secondary structure was predicted (Table S3). The random coil accounted for ~ 30% and the extended strand accounted for ~ 50% of the secondary structure in both willow and poplar. The β-turn had the lowest proportion. The schematic of the secondary structures showed that SpuXTH and PtrXTH proteins contained the conserved E × D × E × domain, acting an active site (Fig. 4). Except for the ancestral and III-A group members, I/II and III-B group had an N-glycosylation domain. Additionally, there are two conserved domains after the conserved E × D × E × domain, loop 2 and loop 3. Loop 2 in I/II, III-B and ancestral group members was shorter than that in III-A group. A conserved WAT × GG sequence (loop 3) of I/II, III-B and ancestral group members or a conserved SWATE × sequence (loop 3) of III-A group members was located in the C-terminal region. The C-terminal region of XTH proteins often contains highly conserved cysteines, which can form disulfide bonds and contribute to the stability of XTHs' protein structure [34].

Six XTH proteins with different amino acids at the activation sites, randomly selected from poplar and willow, were predicted using three-dimensional structure simulations (Fig. 4C, F). The homology modeling method was used to predict the tertiary structure of unknown proteins using homologous protein with known tertiary structure in the database. The results revealed that the six protein models consisted of large β-sheets arranged in a sandwich manner. Differences in amino acids at the activation sites led to subtle differences in tertiary structure and function between the proteins. These proteins are ready for further study.

### Analysis of cis-elements in *XTHs*

**Fig. 5:**
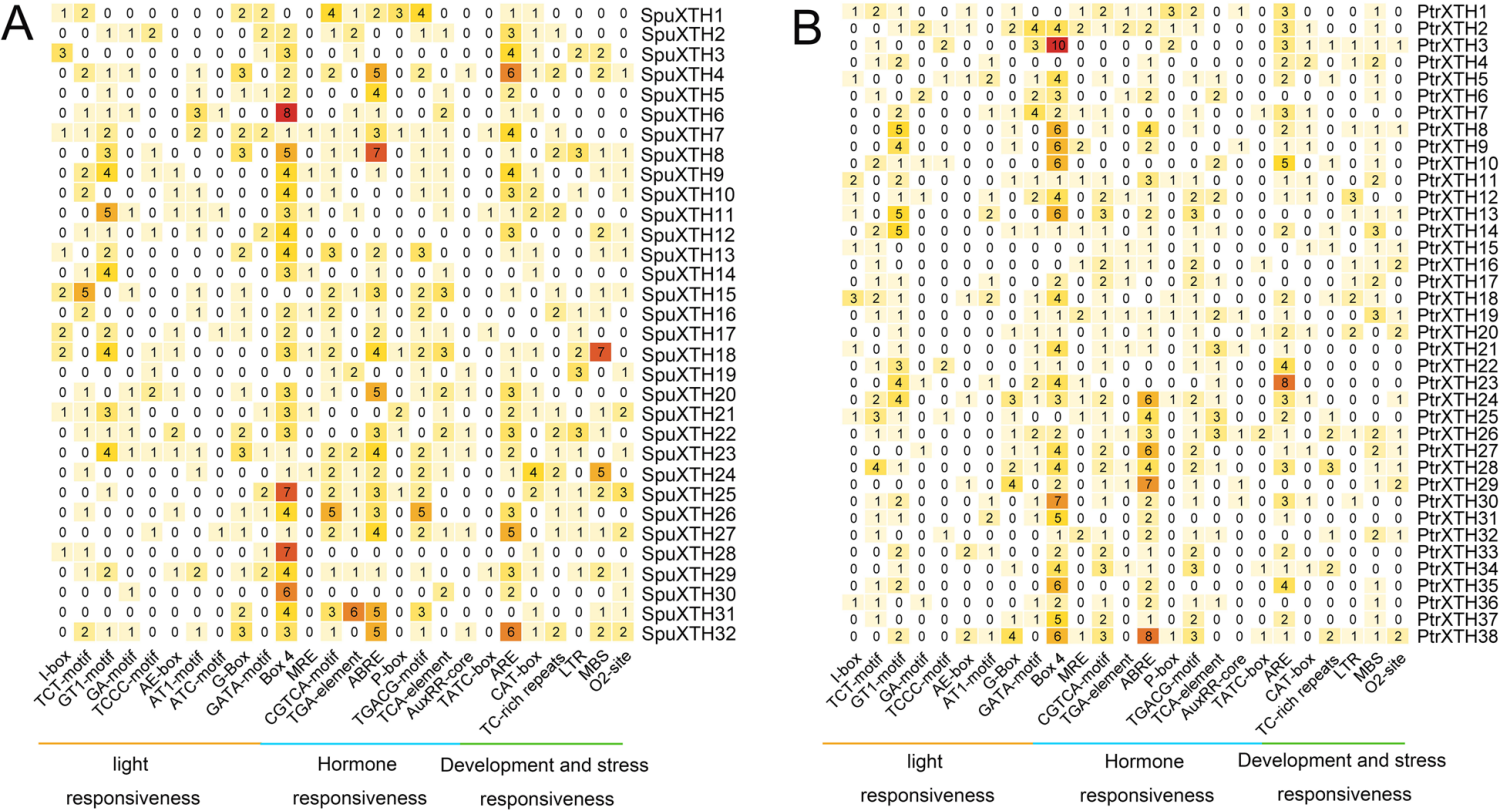
Analysis of cis-elements in *SpuXTH* and *PtrXTH* promoters. **A** Number of distinct cis-elements in each *SpuXTH* genes. **B** Number of distinct cis-elements in each *PtrXTH* genes

The 2000 bp upstream sequence of coding sequence (CDS) was extracted to predict cis-elements in *SpuXTH* and *PtrXTH* genes (Fig. 5). Most of the cis-elements, *e.g*., I-box, TCT-motif, GT1-motif, GA-motif, TCCC-motif, AE-box, AT1-motif, ATC-motif, G-box, GATA-motif, box 4 and MRE, were classified as light-responsive elements. Circadian rhythm control, meristem expression and zein metabolism regulation elements were related to growth and development. Anaerobic induction, defense and stress response, drought induction and low temperature induction elements were related to abiotic stress. Salicylic acid response, methyl jasmonate, abscisic acid, gibberellin and auxin response elements were included in the hormone related elements. Among them, the abundances of auxin and methyl jasmonate elements were contained in *XTH* genes, and these *XTH* genes may be regulated during grafting process.

### Expression pattern of *XTH* genes in grafted plants

**Fig. 6:**
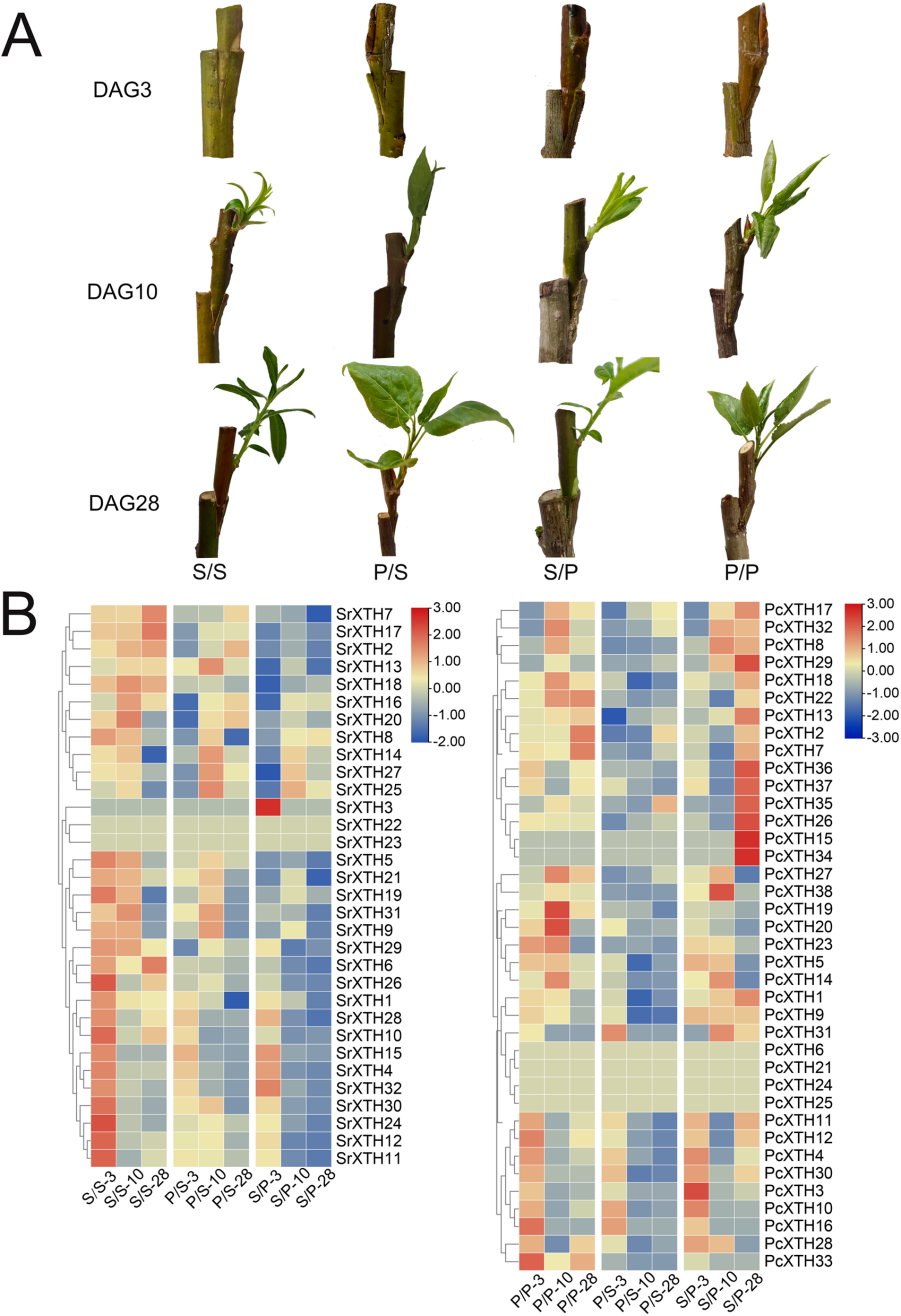
Morphology of four grafted combinations (P/P, P/S, S/S and S/P) and expression patterns of *PcXTH* and *SrXTH* genes. **A** Four grafting combinations of poplar and willow at 3, 10, and 28 days after grafting. **B** Heat map of the expression levels of *SrXTH* genes on the third, tenth and twenty-eighth days after grafting in grafted combinations S/S, P/S and S/P based on FPKM values. Heat map of the expression levels of *PcXTH* genes on the third, tenth and twenty-eighth days after grafting in grafted combinations P/P, P/S and S/P based on FPKM values

We analyzed the expression pattern of *XTH* genes in the four grafted combination: *P. cathayana* grafted onto *P. cathayana* (P/P), *P. cathayana* grafted onto *S. rehderiana* (P/S), *S. rehderiana* grafted onto *P. cathayana* (S/P) and *S. rehderiana* grafted onto *S. rehderiana* (S/S) (Fig. 6). In the self-grafted combinations, *XTH* genes were highly expressed after three and ten days of grafting. Two *SpuXTH* genes (*SpuXTH23* and *SpuXTH33*) and four PtrXTH *(PtrXTH6*, *PtrXTH21*, *PtrXTH24* and *PtrXTH25*) genes didn’t express in four grafted plants. The expression level of most *XTH* genes is higher in self-grafted combinations than in reciprocal-grafted combinations. At three, ten and twenty-eight days, the *PtrXTH* genes were highly expressed in S/P and the *SpuXTH* genes were highly expressed in P/S.

**Fig. 7:**
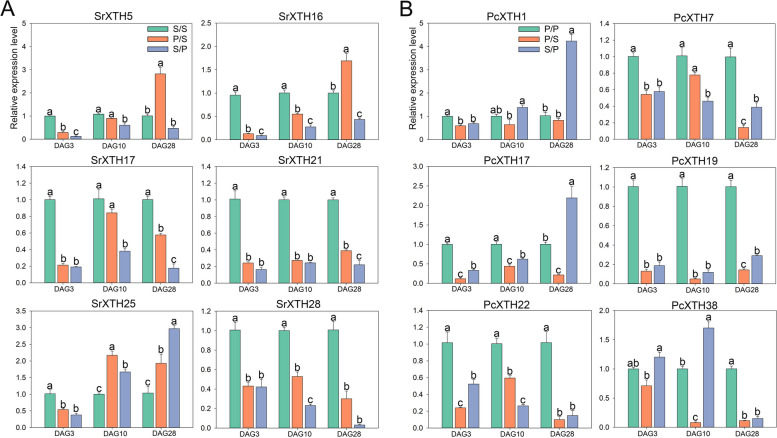
Expression profiles of *SrXTH* and *PcXTH* genes in different grafted combinations after three, ten and twenty-eight days. Error bars on the graph indicate the mean standard deviation for each triplicate treatment

**Fig. 8:**
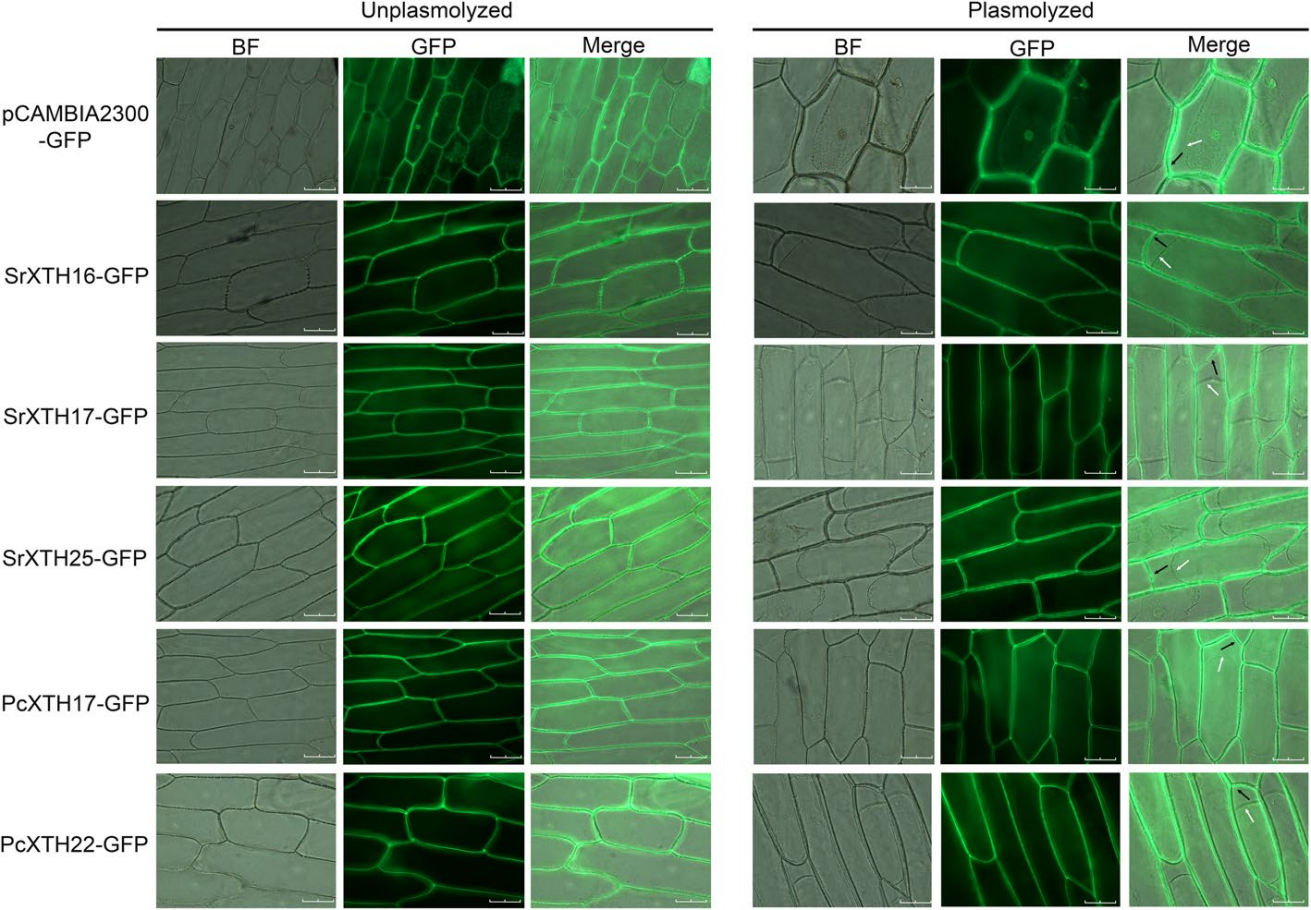
Subcellular localization of the SrXTH16, SrXTH17, SrXTH25, PcXTH17 and PcXTH22 proteins. pCAMBIA2300-GFP (empty vector), pCAMBIA2300-*SrXTH16*, pCAMBIA2300-*SrXTH17*, pCAMBIA2300-*SrXTH25*, pCAMBIA2300-*PcXTH17* and pCAMBIA2300-*PcXTH22* were transiently expressed in onion epidermal cell layers. Scale bar = 50.0 μm

The quantitative real time PCR (RT-qPCR) results showed that compared with S/S, *SrXTH16* and *SrXTH17* had lower expression in P/S and S/P, while *SrXTH25* was highly expressed in P/S and S/P (Fig. 7A). Compared with the P/P, *PcXTH17* and *PcXTH22* had low expression in P/S and S/P during graft healing process (Fig. 7B). To gain their molecular functions, the *SrXTH16*-GFP, *SrXTH17*-GFP, *SrXTH25*-GFP, *PcXTH17*-GFP and *PcXTH22*-GFP plasmids were transferred into *Agrobacterium*. The subcellular results showed that the SrXTH16, SrXTH17, SrXTH25, PcXTH17 and PcXTH22 proteins were located on the cell wall (Fig. 8).

### Prediction of transcription factors that regulated *XTH* genes

**Fig. 9:**
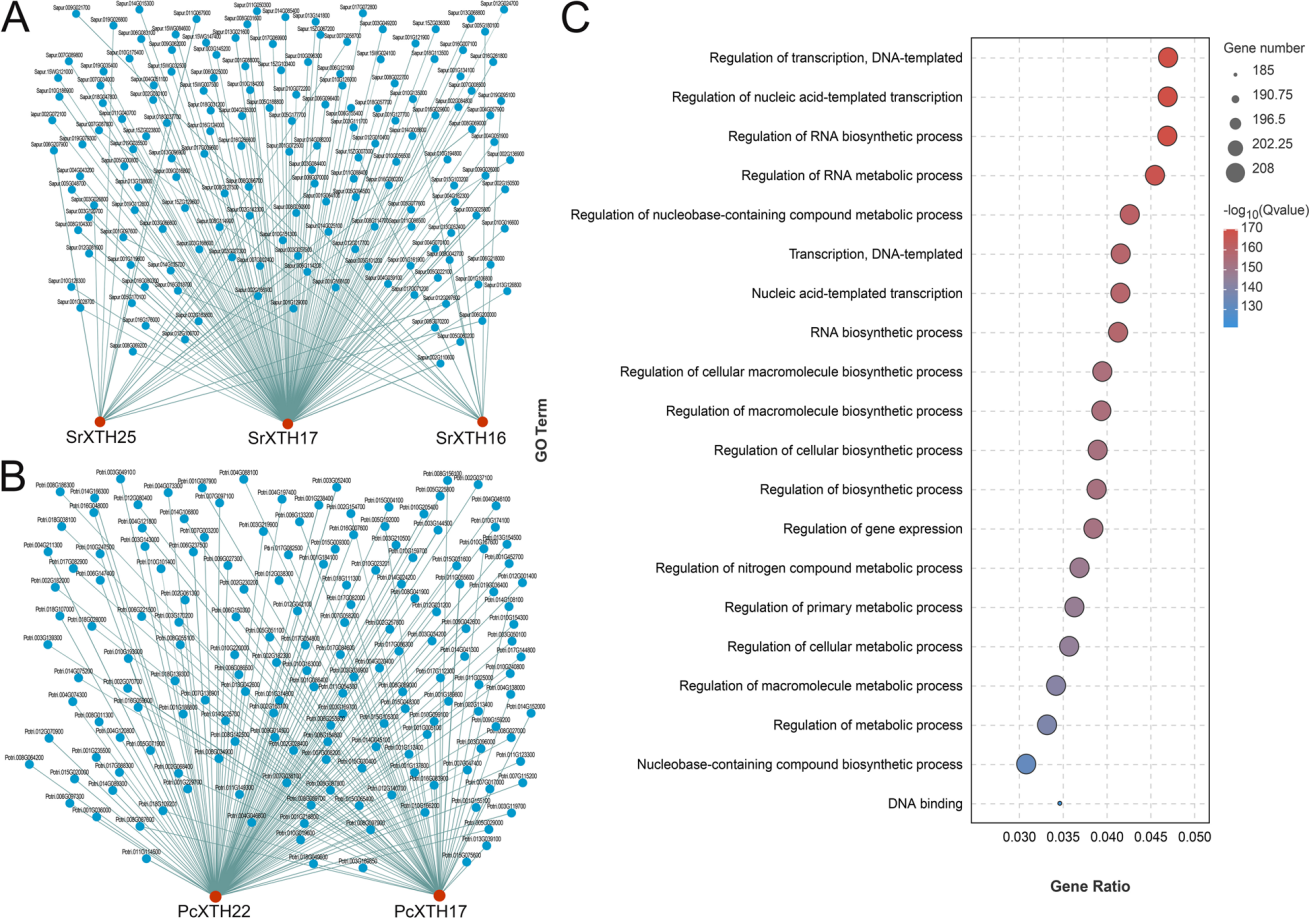
Predicted transcription factors that regulate *XTH* genes and GO analysis of the selected transcription factors. **A** and (**B**) There are 323 pairs of interacting proteins for 5 *XTH* genes. The red circle represents the *PcXTH* or *SrXTH* genes. The blue circle represents the transcriptional factor in each clade. The thickness of the green line indicates the reliability of the predicted results. The line is thicker, the result is more reliable

A total of 323 transcription factors were predicted to regulate *SrXTH16*, *SrXTH17*, *SrXTH25*, *PcXTH17* and *PcXTH22* (Fig. 9A and B). They were 13 of *SrXTH16*, 125 of *SrXTH17*, 19 of *SrXTH25,* 60 of *PcXTH17* and 106 of *PcXTH22*. All these *XTH* genes were predicted to be regulated by members of the NAC, DOF (DNA binding with one finger), HD-Zip (homeodomain-leucine zipper) and MYB (v-myb avian myeloblastosis viral oncogene homology) transcription factor families, such as MYB6 (Potri.004G088100), DOF1.4 (Potri.011G055600), HAT4 (Potri.014G045100) and NAC017 (Potri.002G061300). *PcXTH17*, *SrXTH17* and *SrXTH25* were regulated by ethylene responsive factor (ERF) transcription factor family, *e.g*., ERF016 (Potri.018G038100), ERF106 (Sapur.001G064100) and DREB3 (Sapur.003G026800). *PcXTH17* and *SrXTH17* genes were regulated by the auxin response factors (ARF), *e.g*., ARF19 (Sapur.006G114200) and ARF2A (Potri.015G105300). Then we performed Gene Ontology (GO) enrichment analysis on predicted transcription factors (Fig. 9C). In addition to the functions related to gene regulation, 208 transcription factors were also involved in cellular biosynthetic process and cellular metabolic biosynthesis process. These indicated that these *XTH* genes may participate in the graft healing process by regulating cell division and growth.

## Discussion

Poplar grafted to willow has a higher successful rate than willow grafted to poplar [8]. However, the molecular mechanism is not clear. XTH proteins are a kind of xyloglucan transferases/hydrolases involved in the regulation of cell wall relaxation. Due to their roles in cell wall modification, we believe that the *XTH* gene may have important effects on the grafted healing.

38 and 33 *XTH* genes were respectively identified in poplar and willow. They were divided into four groups: I/II, III-A, III-B and ancestral group. The XET proteins belonging to the I/II and III-B groups have a conserved N-linked glycosylation site, which is thought to be important for protein stability [19, 29, 35]. N-glycosylation domain was absent almost in all III-A and ancestral group members, which was consistent with our results [36, 37]. Therefore, the XTH proteins of I/II and III-B group have higher protein stability than that of III-A and ancestral group. XTH proteins belonging to I/II, III-B and ancestral group have XET activity, while XTH proteins belonging to III-A group have XEH activity [38]. The length of loop 2 was the key point that determined the XET or XEH activity of XTH proteins. The loop 2 of I/II, III-B and ancestral group is shorter than that of III-A group proteins [29, 37]. XET proteins specifically recognizes xyloglucan and forms new xyloglucan chains to expand call walls [39]. Previous research found that high expression of β-1,4-glucanase make tobacco successfully graft with 73 species of plants [28]. Evidence shows that it plays an important role in the grafting process of tobacco by facilitating cell wall reconstruction near the graft interface [28]. Because XETs can expand cell wall to promote cell wall construction, we believe that XET enzymes play an important role in grafting process.

Heatmap results showed that some genes didn’t express and some genes highly expressed during graft healing process. Phylogenetic analysis showed that the *SpuXTH6* and *PtrXTH6* were assigned to the ancestral group. A recent study reported that genes in the ancestral group were not the ancestors of the other group genes, but diverged after other groups, possibly introducing some new roles in flowering plants [26]. They were predominantly or exclusively expressed in reproductive tissues of flowering plants but not found in any lower plants [26]. This may be the reason that the expression levels of *SrXTH6* and *PcXTH6* were not detected in stem tissue. *SpuXTH22*, *SpuXTH23*, *PtrXTH24* and *PtrXTH25* belonged to I/II group. *AtXTH15*, which was homologous to them, was related to shade and mainly expressed in seeds [40]. *PtrXTH21* belonged to III-B group. *AtXTH27*, which was homologous to it, regulated tracheary element development [41]. The *SpuXTH16*, *SpuXTH25* and *PtrXTH17* belonged to I/II group, while *SpuXTH17* and *PtrXTH22* belonged to III-B group. They highly expressed in the stem. *AtXTH9*, homologous to *SpuXTH16*, *SpuXTH25* and *PtrXTH17*, regulated secondary call wall formation [42]. The subcellular localization results indicated that SrXTH16, SrXTH17, SrXTH25, PcXTH17 and PcXTH22 proteins were located on cell walls.

Many genes encode cell wall modification enzymes play important roles during graft healing process [11]. Genes encoded cell wall-plasma membrane linker proteins and cell wall LRR proteins and genes related to phloem development and cellulose biosynthesis were up-regulated at 3-28 days after grape self-grafted plants [12]. *AtXTH28* highly expressed after *Arabidopsis* and tobacco grafted combination [28]. Our results also found that *SrXTH16*, *SrXTH17*, *SrXTH25*, *PcXTH17* and *PcXTH22* highly expressed at 3-28 days after poplar and willow self-grafted combinations. The up-regulated *XTH* genes may lead to the accumulation of xyloglucan in the cell wall of tomato graft interface to promote cell adhesion [14]. Cell wall modification related genes actively expressed, causing significant changes in the cell wall to promote the formation of secondary plasmodesmata at the grafted junction [43]. It provides energy for rootstocks and promotes the material exchange between rootstocks and scions [43]. These are key points to improve the survival rate of grafting [44]. The high survival rate of reciprocal grafted plants is also related to the positive expression of cell wall related genes [45, 46]. Our results found that the expression levels of *PcXTH22* and *PcXTH17* in the low survival rate grafted combination S/P were higher than those in the high survival rate grafted combination, which may play an important regulatory role in the healing process of S/P grafting. *SrXTH16*, *SrXTH17* and *SrXTH25* have higher expression levels in P/S than in S/P, which may be important genes affecting the survival rate of reciprocal graft between poplar and willow.

The expression levels of *XTH* genes are regulated by transcription factors [27, 47]. They binding with cis-elements of genes is one of the important mechanisms regulating expressions of genes [48–50]. Based on this fact and gene co-expression network, we predicted transcription factors that regulated five *XTH* genes: *SrXTH16*, *SrXTH17*, *SrXTH25*, *PcXTH17* and *PcXTH22*. Results showed that all five *XTH* genes were regulated by NAC, MYB and DOF transcription factors. NAC transcription factors, *e.g.*, NST3/ANAC012, NST1/ANAC043, PtrWND2 and PtrWND6, activate plant secondary cell walls growth program resulting in ectopic deposition of secondary cell walls [47, 51]. The *AtXTH10* was directly regulated by NST3 and VND7 [47]. In our study, *PcXTH22* was regulated by PtrNAC017(Potri.002G061300). Previous study found that ANAC071, homologous with PtrNAC071, bound to the *AtXTH19* and *AtXTH20* promoters to induce their expression in the distal part of an incised stem and their involvement in cell proliferation in the tissue reunion process [27]. MYB transcription factors, *e.g*., MYB46, MYB83 and MYB103 were regulated by secondary wall NAC transcription factors to participate in the biosynthesis of cell walls [47, 52]. In self-grafted pepper plants, MYB86 was predicted to regulate *XTH22* and *XTH38* [53]. Our study found that PcMYB61 (Sapur.005G000800) regulated *SrXTH25*. AtMYB46, which was homologous with PtrMYB61, can activate the synthesis of cellulose and hemicellulose [54]. DOF transcription factors participates in regulating tissue differentiation and vascular system development [50]. Overexpression of AtDOF5.4 in *Arabidopsis* plants exhibited dwarfing phenotypes [55]. AtDOF5.8 was thought to play important role in the early stages of primitive meristem cell formation and vascular system formation [50]. In our results, *PcXTH17* was regulated by PcDOF1.2 (Potri.002G070700). The AtDOF2.1 transcription factor, which is homologous to DOF1.2, was induced after injury and involved in cell wall modification [56]. Existing grafting experiments showed that four DOF transcription factors (HCA2, TMO6, DOF2.1, and DOF6) mutant plants inhibited phloem reconnection after grafting, while overexpressing plants accelerated phloem reconnection after grafting [56]. Therefore, we thought that the NAC, MYB, and DOF family transcription factors may participate in the grafting healing process by regulating the *XTH* gene.

## Conclusions

In this study, 38 *PtrXTH* genes and 32 *SpuXTH* genes were identified, and they were divided into three subfamilies. Tandem duplication contributed most to the expansion of the *PtrXTH* family. Five *XTH* genes (*SrXTH16*, *SrXTH17*, *SrXTH25*, *PcXTH17* and *PcXTH22*) are thought to play important roles in Salicaceae grafting and are regulated by some transcription factors (NAC, MYB and DOF) during graft healing process. In summary, this study improved our understanding of the *PcXTH* and *SrXTH* gene families and lay the foundation for further exploration on grafting in poplar and willow.

## Methods

### Identification of* XTH* family genes

Gene, protein and gene annotation files of *P. trichocarpa* and *S. sinopurpurea* were downloaded from the Phytozome website (https://phytozome-next.jgi.doe.gov/). Meanwhile, the protein and gene sequences of 33 *XTH* family genes identified in *Arabidopsis* were also downloaded from Phytozome [57].

Two methods were used to identify *XTH* family genes in Salicaceae. To obtain profiles of XTH protein domains, the query numbers PF00722 and PF06955 found in literatures were used to search in the pfam-A database (http://pfam.xfam.org/), which was derived from the pfam website [24]. Using the query results, the Simple Hidden Markov Model (HMM) Search function in TBtools software could retrieve all potential XTH family members from whole genome sequences in *P. trichocarpa* and *S. sinopurpurea* [58]. The threshold used for identifying the XTHs domains was 1E-5. Another way was to perform a two- way blast alignment using the Two Sequence Files tool in TBtools in combination with the protein sequences of Arabidopsis *XTH* family genes and whole genome sequences of *P. trichocarpa* and *S. sinopurpurea* to identify all potential *XTH* family genes in Salicaceae [58]. To further screen the *XTHs*, the Batch SMART function of TBtools software was used to visualize the protein domains of *XTHs* obtained separately by the two methods [58, 59]. After removing the domain mismatch and deficiency, 32 *SpuXTHs* and 38 *PtrXTHs* were identified (Table S4).

### Physical and chemical analysis and the secondary and tertiary structure of proteins

The length, molecular weight, theoretical isoelectric point, instability index, aliphatic index and grand average of hydropathicity of XTH proteins were characterized by using the online tool ExPASy ProtParam (https://web.expasy.org/protparam/) [60]. The secondary structure of XTH proteins was predicted by the online tool SOPMA (https://npsa-prabi.ibcp.fr/cgi-bin/npsa_automat.pl?page=npsa%20_sopma.html). Finally, the online tool SWISS-Model Interactive Workespace (https://swissmodel.expasy.org/interactive) was used to predict the tertiary structure of XTH proteins by homolog modeling method.

### Analysis of gene structure, protein conserved motif and protein conserved domain

The conserved motif profiles of the proteins were predicted by MEME Suite 5.4.1 (https://meme-suite.org/meme/tools/meme) using default parameters, and the motif number was set to 15 for poplar and willow [61]. The profiles of conserved protein domains were predicted and listed by Batch SMART tool of TBtools software. Then, the evolutionary tree, gene structure, domain, and protein motif were draw using Gene Structure View tool of TBtools software.

### The collinearity analysis, structural-based sequence alignment and phylogenetic analysis

The One step MCScan X tool in TBtools software was used for the collinearity analysis [58]. The secondary structures of PttXET16-34 (PDB id:1UN1) and TmNXG1 (PDB id: 2UWA) were downloaded from the PDB database (https://www.rcsb.org/) and used as a comparison standard [62]. The Align by Clustal W tool with default parameters in MEGA11.0 was used for multiple sequence alignment with downloaded data and protein sequences of poplar and willow [63, 64]. The multiple sequence alignment results were analyzed and visualized using ESPript 3.0 version (https://espript.ibcp.fr/ESPript/ESPript/index.php) to predict the secondary structures of SpuXTHs and PtrXTHs. The phylogenetic tree was constructed by neighbor joining method using multiple sequence alignment. The bootstrap value was set at 1000. The evolutionary tree was modified by Evolview3.0 online software [64].

### Calculation of Ka/Ks

The Ka, Ks and their ratio of *XTH* pairs were calculated using the Simple Ka/Ks Calculator function of TBtools software [58]. Ks is the calculated value of synonymous mutation frequency and Ka is the calculated value of non-synonymous mutation frequency [65]. The ratio of Ka/Ks can be used to estimate the selection pressure. If the ratio is greater than 1, it means positive selection [66]. And if the ratio is less than 1, it indicates purity selection [66]. If the ratio is the equal to 1, it means neutral selection [66].

### Analysis of cis-acting elements

Firstly, using TBtools’ GTF/GFF3 Sequences extractor function to extract 2000 bp upstream of the CDS sequences of *XTH* family genes, then predicting cis-acting elements using Plant Care database (http://bioinformatics.psb.ugent.be/webtools/plantcare/html/). The results were visualized by HeatMap tool of TBtools software.

### Plant materials and transcriptome analysis

Four grafting combinations (scion/rootstock: P/P, S/S, P/S, S/P) were made using *P. cathayana* (P) and *S. rehderiana* (S). After three, ten and twenty-eight days of grafting, callus was collected from the grafted healing sites (five centimeters each from the contact area of the scion and rootstock). Fifteen grafted plants were performed at each time point, and nine grafted plants were randomly selected and divided into three groups for callus collection. Samples collected at three, ten and twenty-eight days were placed into liquid nitrogen. Total RNA was extracted using Trizol reagent kit (Invitrogen, Carlsbad, CA, USA) following the manufacturer’s protocol. The Agilent 2100 Bioanalyzer (Agilent Technologies, Palo Alto, CA, USA) was used to assess RNA quality and the RNase free agarose gel electrophoresis was used to verify the results. After total RNA extraction, eukaryotic mRNA was enriched by Oligo(dT) beads, and prokaryotic mRNA was enriched by removing rRNA by Ribo-ZeroTM Magnetic Kit (Epicentre, Madison, WI, USA). Then the enriched mRNA was fragmented into short fragments using fragmentation buffer and reverse transcribed into cDNA by using NEBNext Ultra RNA Library Prep Kit for Illumina (NEB #7530, New England Biolabs, Ipswich, MA, USA). The purified double-stranded cDNA fragments were end repaired, A base added, and ligated to Illumina sequencing adapters. The ligation reaction was purified with the AMPure XP Beads (1.0X), and amplified by polymerase chain reaction (PCR). The cDNA library was sequenced using Illumina Novaseq6000 by Gene Denovo Biotechnology Co. (Guangzhou, China).The paired-end clean reads were mapped to the reference genomes (*P. trichocarpa* and *S. purpurea* genomes). For each transcription region, a FPKM (fragment per kilobase of transcript per million mapped reads) value was calculated to quantify its expression abundance and variations.

### Experimental validation of *XTH* transcript levels by RT-qPCR analysis

After three, ten and twenty-eight days of grafting, the grafted healing sites were collected and frozen in liquid nitrogen. All samples were then stored at -80 °C. Total RNA was extracted using a RNeasy kit (Tiangen, Biotech) according to the instructions. Total RNA (1 μg) was treated with a FastQuant RT kit (with gDNase; Yeasen, https://www.yeasen.com/) to get reverse transcript. *18S ribosomal RNA* (*rRNA*) was used as the reference gene [67]. The primer sequences presented in Table S5 were designed by Primer Premier 6.0 and blasted in NCBI. RT-qPCR analysis was performed using LineGene 9600 Plus platform (Bio-er, Hangzhou). The transcript levels were analyzed using the 2^−ΔΔCt^ method and means ± standard errors (SE) [68].

### Subcellular localization of the XTH proteins

The CDS of *SrXTH16*, *SrXTH17*, *SrXTH25*, *PcXTH17* and *PcXTH22* were amplified by PCR. Link PCAMBIA2300-GFP vector was linked to the amplification products by homologous recombination method. The linked products were first introduced into DH5α-competent cells to screen for plasmids with correct sequences, which were then introduced into *Agrobacterium tumefaciens *GV3101 strain for transient expression in the epidermal cell layer of onion bulbs that incubated on medium for 1 day in advance. Infected onion epidermal cell layers were maintained at 25 °C in the dark and harvested for fluorescence examination 48 h after infection. GFP signals in the transiently infected onion epidermis were observed using a Leica DM4 B upright fluorescence microscope (Leica, Germany). A 30% sucrose solution was used to separate onion cytoplasmic walls. The relevant primers are listed in supplementary Table S5.

### Prediction of transcription factors that regulated *XTH* genes

The potential transcription factors that regulated five *XTH* genes (*SrXTH16*, *SrXTH17*, *SrXTH25*, *PcXTH17* and *PcXTH22*) were predicted using the transcription factor targeting analysis tool on the GENE DENOVO cloud platform (https://www.omicsmart.com/index.php). Pearson correlation analysis was performed using the expression levels of *XTH* genes and transcription factors in R software using the readxl (version 1.4.3) and stringr (version 1.5.0) packages, and transcription factors with correlation coefficients greater than or equal to 0.8 were screened for plotting on the GENE DENOVO cloud platform (Table S6). In addition, GO analysis of the selected transcription factors was performed on the Omicsmart online platform ((https://www.omicsmart.com/). The *P* value ＜0.01. 

### Supplementary Information

**Additional file 1: Supplementary Table 1.** The characteristics of PtrXTHs and SpuXTHs. **Supplementary Table 2. **The Ka/Ks ratios of poplar and willow gene pair. **Supplementary Table 3.** The secondary structure prediction of PtrXTHs and SpuXTHs. **Supplementary Table 4.** The protein sequences of poplar and willow. **Supplementary Table 5.** Specific primers for *XTH* genes in poplar and willow. **Supplementary Table 6.** The predicted transcription factors that regulate five *XTH* genes.

## Data Availability

The data reported in this paper have been deposited in the OMIX, China National Center for Bioinformation / Beijing Institute of Genomics, Chinese Academy of Sciences (https://ngdc.cncb.ac.cn/omix/release/OMIX004709)*.*

## Abbreviations

ARF: Auxin response factor
CDS: Coding sequence
DOF: DNA binding with one finger
GH16: Glycoside hydrolase family
GO: Gene Ontology
HD-Zip: Homeodomain-leucine zipper
HMM: Hidden Markov Models
JA: Jasmonic acid
Ka: Synonymous substitution
Ks: Non-synonymous substitution
MeJA: Methyl jasmonate
MW: Molecular weight
MYB: V-myb avian myeloblastosis viral oncogene homology
NAC: NAC domain containing protein
pI: Isoelectric point
UTRs: Untranslated regions
WIND1/2/3: Wound induced dedifferentiation 1/2/3
WOX4: WUSCHEL-related homeobox 4
XEH: Xyloglucan endoglucosidase
XET: Xyloglucan endohydrolase
XTH: Xyloglucan endotransglucosylase/hydrolase
